# (18F)FDG-PET brain imaging during the micturition cycle in rats detects regions involved in bladder afferent signalling

**DOI:** 10.1186/s13550-015-0132-0

**Published:** 2015-10-15

**Authors:** Yves Deruyver, Roma Rietjens, Jan Franken, Silvia Pinto, Ann Van Santvoort, Cindy Casteels, Thomas Voets, Dirk De Ridder

**Affiliations:** Laboratory of Experimental Urology, Department of Development and Regeneration, KU Leuven, Herestraat 49, 3000 Leuven, Belgium; Laboratory of Ion Channel Research, Department of Cellular and Molecular Medicine, KU Leuven, Leuven, Belgium; Division of Nuclear Medicine, Department of Imaging and Pathology, KU Leuven, Leuven, Belgium; TRP Channel Research Platform Leuven (TRPLe), KU Leuven, Herestraat 49, 3000 Leuven, Belgium

**Keywords:** Brain imaging, Urinary bladder, Rats, Brain bladder control, Small-animal PET

## Abstract

**Background:**

This feasibility study established an experimental protocol to evaluate brain activation patterns using fluorodeoxyglucose positron emission tomography ((18F)FDG-PET) during volume-induced voiding and isovolumetric bladder contractions in rats.

**Methods:**

Female Sprague-Dawley rats were anaesthetized with urethane and underwent either volume-induced voiding cystometry or isovolumetric cystometry and simultaneous functional PET brain imaging after injection of (18F)FDG in the tail vein. Brain glucose metabolism in both groups was compared to their respective control conditions (empty bladder). Relative glucose metabolism images were anatomically standardized to Paxinos space and analysed voxel-wise using Statistical Parametric Mapping 12 (SPM12).

**Results:**

During volume-induced voiding, glucose hypermetabolism was observed in the insular cortex while uptake was decreased in a cerebellar cluster and the dorsal midbrain. Relative glucose metabolism during isovolumetric bladder contractions increased in the insular and cingulate cortices and decreased in the cerebellum.

**Conclusions:**

Our findings demonstrate that volume-induced voiding as well as isovolumetric bladder contractions in rats provokes changes in brain metabolism, including activation of the insular and cingulate cortices, which is consistent with their role in the mapping of bladder afferent activity. These findings are in line with human studies. Our results provide a basis for further research into the brain control of the lower urinary tract in small laboratory animals.

## Background

Storage of urine and micturition are controlled by a complex neural circuitry, located in the brain, spinal cord and peripheral ganglia and nerves [1]. In the last decade, it has become clear that the brain plays a crucial role in normal voiding control and is also involved in lower urinary tract dysfunction (LUTd). Vice versa, LUTd has a disruptive impact on cortical activity leading to neurobehavioral consequences such as hyperarousal and sleep disturbance [2]. The brain does not merely initiate micturition by activating efferent signalling pathways resulting in detrusor contraction but is also constantly receiving input from the lower urinary tract (LUT) via afferent pathways originating in the urothelium and small afferent nerves in the bladder wall. Today, it is well established that alterations in this sensory system may also lead to LUTd [3].

The advent of brain imaging techniques such as functional magnetic resonance imaging (fMRI) and positron emission tomography (PET) has encouraged research into the brain control of the bladder. Although the number of brain imaging studies is still relatively small, they have already provided us with valuable new insights. Activation of the insular cortex and anterior cingulate cortex (ACG) during urine storage is a very common finding. Neuroanatomical and electrophysiological studies show that they play a role in the mapping of visceral sensations and autonomic motor control [4]. Activation of the pontine micturition centre (PMC) is necessary for the initiation of micturition, as demonstrated almost a century ago in a cat by Barrington [5]. Involvement of other areas such as the prefrontal cortex, cerebellum and (hypo)thalamus has been less consistently shown, and their role remains elusive [6].

Recently, functional brain imaging studies in rats using fMRI during cystometry have detected several of these brain regions during different phases of the micturition cycle, proving that it is feasible to study the supraspinal control of the LUT in small-animal models [7, 8]. However, up to date, no data on small-animal PET imaging of the brain related to LUT control is available in literature. PET imaging can confirm and complement the results obtained by fMRI in rodents during the micturition cycle, and it can be a valuable model for translational studies. Brain imaging techniques have the potential to reveal important new information about the neural control of the LUT, since they enable studying supraspinal bladder control in both humans and laboratory animals during the different phases of the micturition cycle. Not only can they contribute to our understanding of normal LUT physiology, they can also be valuable for a better understanding and treatment of LUTd. Different studies have already provided evidence for a link between aberrant brain processing and LUTd in patients with urge incontinence, Fowler’s syndrome and Parkinson’s disease [9].

In the present feasibility study, we established an experimental protocol to evaluate brain metabolism during volume-induced voiding and isovolumetric bladder contractions in anaesthetized rats using small-animal PET brain imaging with the aim of providing a basis for further PET-based research in this field.

## Methods

### Animals

Experiments were conducted on 11–12-week-old female Sprague-Dawley rats weighing 245–289 g. Animals were housed under standard laboratory conditions with a 12-h light-dark cycle and free access to food pellets and tap water. All experiments were approved by the KU Leuven Ethical Committee for laboratory animals under project number P092/2014.

### Volume-induced voiding and isovolumetric cystometry

Volume-induced voiding cystometry and isovolumetric cystometry were performed as previously described [8]. Briefly, rats were anaesthetized with a subcutaneous injection of urethane (1.3 g/kg). Subsequently, a PE-50 polyethylene catheter (Instech Laboratories, PA, USA) was inserted in the bladder dome and fixed with a purse-string suture. In the isovolumetric cystometry group and its controls, the urethra was ligated just underneath the bladder neck with a 5/0 monofilament suture. Finally, the abdominal wall and skin were closed and the animal was transferred to the small-animal PET imaging facility.

### Positron emission tomography imaging combined with cystometry

The pressure transducer (Biopac Systems, CA, USA) and infusion pump (KR Analytical, UK), which were connected to the bladder catheter via a three-way valve, were positioned directly adjacent to the PET scanner. The pressure transducer was recorded using a data acquisition system (Acknowledge, Biopac Systems, CA, USA). Rats were installed in supine position on a thermal pad in the small-animal PET. For volume-induced voiding, a saline infusion rate of 200 μl/min was used. For isovolumetric cystometry, bladders were filled with saline at a constant rate of 100 μl/min until isovolumetric bladder contractions were induced. If bladder contractions were not maintained, additional saline was infused. Subsequently, tail veins were catheterized for injection of fluorodeoxyglucose ((18F)FDG). (18F)FDG was prepared using an Ion Beam Applications synthesis module. On average, 25.4 ± 0.5 MBq of (18F)FDG was injected in 500 μl of saline. (18F)FDG acquisitions were started 90 min after urethane anaesthesia dynamically for 90 min (frame duration 4 × 15 s, 4 × 60 s, 5 × 180 s, 8 × 300 s, 3 × 600 s) immediately after tracer injection. Small-animal PET imaging was performed using a FOCUS 220 positron emission tomograph (Siemens/Concorde Microsystems, TN, USA), which has a transaxial resolution of 1.35 mm full width at half maximum. Data were acquired in a 128 × 128 × 95 matrix with a pixel width of 475 μm. Acquisition rationale and kinetics of the isotope in rats have been described previously [10]: (18F)FDG uptake steadily increases up to 60 min after injection until it reaches a steady state, so that stimuli during these 60 first minutes are characteristic for differences in (18F)FDG uptake.

**Fig. 1:**
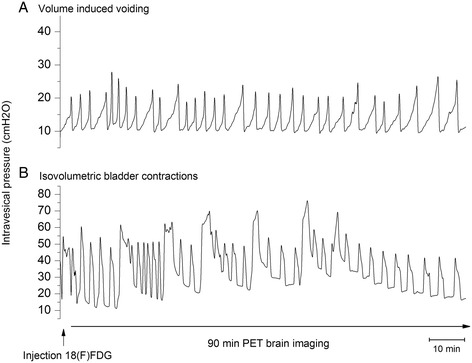
Representative cystometrograms of **a** volume-induced voiding and **b** isovolumetric bladder contractions during a 90-min (18F)FDG-PET brain imaging

To investigate functional brain responses during the micturition cycle, we tested two different experimental settings (Fig. 1). In the first experimental setting, volume-induced voiding cystometry was performed, in which continuous bladder filling and voiding was induced during (18F)FDG-PET brain imaging. Twenty female Sprague-Dawley rats were equally divided into two different groups, a group with continuous infusion of saline at a 200 μl/min filling rate and thus continuous bladder filling and emptying (Fig. 1a) and a second group without infusion of saline (control group). In the second experimental setting, the urethra was ligated just underneath the bladder neck, in order to detect changes in brain metabolism during isovolumetric bladder contractions, a protocol which has previously been used in two fMRI studies in rats [7, 8]. Twelve rats were divided equally into two conditions, a group with intravesical infusion of saline until isovolumetric bladder contractions were induced (Fig. 1b) and a group without infusion (control group).

### Image processing and PET data analysis

For quantification purposes, PET scans were reconstructed using filtered back projection. Parametric images based on standard uptake values (SUV) (=activity concentration (MBq/ml)/injected dose (MBq)) were generated as measure of absolute (18F)FDG uptake. No significant differences in weight or injected activity were present between groups (data not shown). For analysis, we investigated relative (18F)FDG uptake when steady state was reached (3600–5400 s). Relative regional glucose metabolism was determined by normalizing (18F)FDG data to the whole-brain uptake. Predefined volume of interest (VOI) analysis was performed based on a MRI-based functional template of the rat brain in Paxinos standard space, as described by Casteels et al. [10]. Statistical comparison was performed using an unpaired two-sample *t* test. A *p* value of 0.05 or lower was considered significant.

To obtain maximal use of image information, images were analysed on a voxel-by-voxel basis using Statistical Parametric Mapping 12 (SPM12, Welcome Department of Cognitive Neurology, UK). For spatial normalization, individual PET data were normalized using affine transformations to custom-made rat brain PET templates in Paxinos stereotactic space [10]. This allows reporting of results in coordinates directly corresponding to the Rat Brain Paxinos coordinate system. For SPM analysis, data were smoothed with an isotropic Gaussian kernel of 1.2 mm and analysed in a two-sample *t* test. To minimize false-positive findings, *T* maps were interrogated at a peak voxel threshold of *p*_height_ = 0.005 (uncorrected for multiple comparisons) and extent threshold *k*_E_ > 200 voxels (1.6 mm^3^), unless smaller clusters were neurobiologically plausible and relevant in the light of a priori knowledge (smaller clusters are indicated with a superscript letter a in Table 2).

### Conventional statistics

Conventional statistics were carried out using GraphPad Prism 5.1 (Graphpad Software, CA, USA). Data are presented as mean ± SEM. Figures were generated with Origin 8.6 software (OriginLab, MA, USA).

## Results

### Cystometric measurements

Mean intercontractile interval during volume-induced voiding was 102.2 ± 12.68 s. Mean amplitude of voiding contractions was 26.01 ± 2.39 cmH_2_O, mean basal pressure was 12.55 ± 1.36 cmH_2_O and average intravesical pressure during the 90-min scan was 17.67 ± 1.93 cmH_2_O. No cystometric data from the control condition were acquired since the catheter was not connected to the pressure transducer to allow continuous drainage of urine (so intravesical pressure would be 0 cmH_2_O).

In the second experiment (isovolumetric bladder contractions vs. empty bladder), mean intravesical pressure during PET imaging in the isovolumetric bladder contraction group was 39.3 ± 1.9 cmH_2_O. In the empty bladder condition, intravesical pressure was again not recorded since the catheter was not connected to allow continuous drainage of urine.

### Brain glucose metabolism during volume-induced voiding

**Fig. 2:**
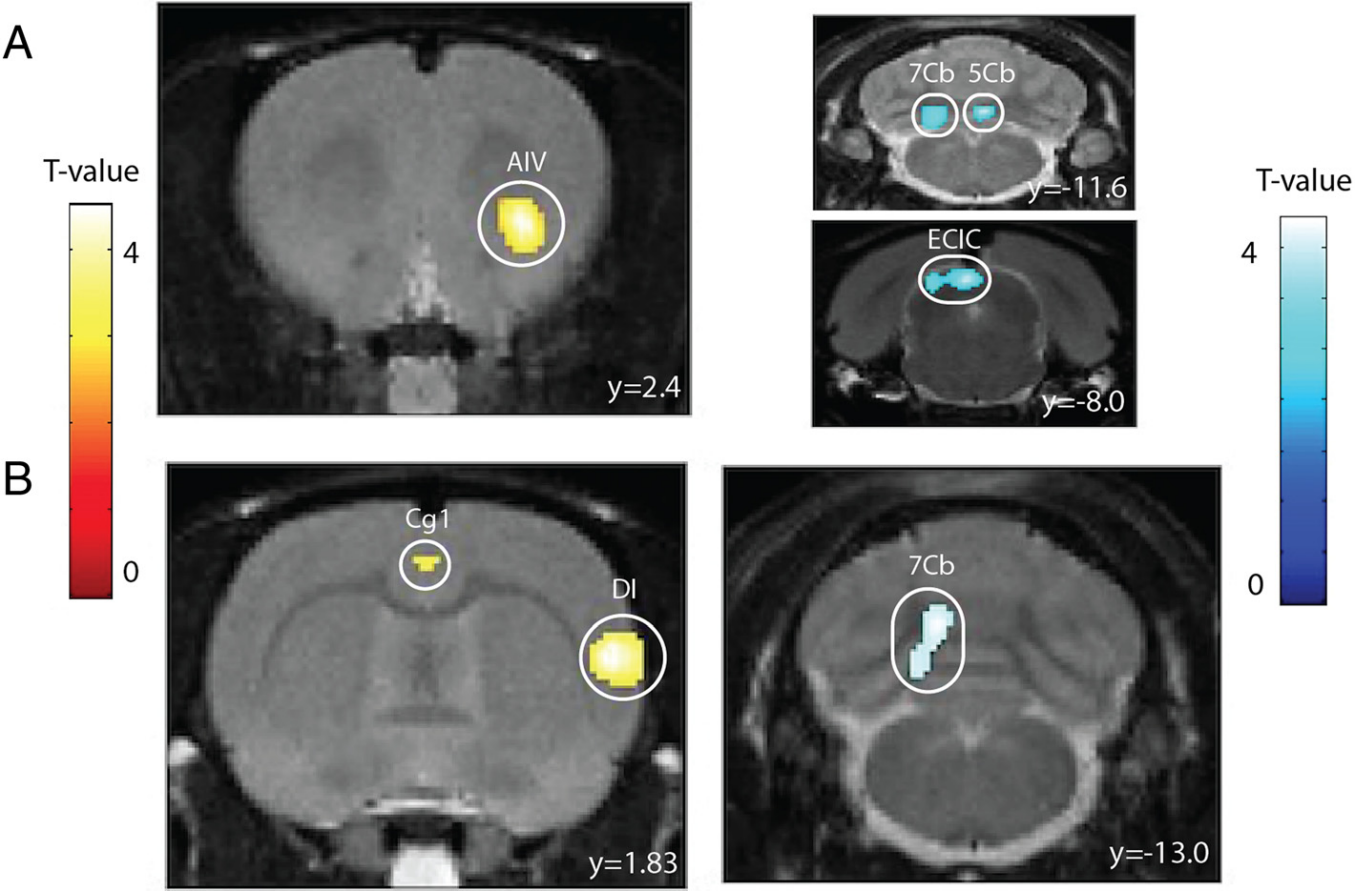
Coronal brain sections with overlay of clusters with increased relative (18F)FDG uptake (*yellow-red*) and decreased relative (18F)FDG uptake (*blue*). **a** Volume-induced voiding (*n* = 10) vs. empty bladder (*n* = 10). **b** Isovolumetric bladder contractions (*n* = 6) vs. empty bladder (*n* = 6). *AIV* agranular insular cortex, *7Cb* 7th cerebellar lobule, *5Cb* 5th cerebellar lobule, *ECIC* external cortex inf colliculus, *Cg1* cingulate cortex, *DI* dysgranular insular cortex

To investigate brain control of the LUT during continuous bladder filling and voiding, we compared brain glucose metabolism between the volume-induced voiding group and its control group. Predefined VOI analysis in Paxinos space showed a significant glucose hypermetabolism in the temporal cortex during continuous bladder filling and voiding (+3.4 ± 1.4 %, *p* = 0.023). SPM12 analysis confirmed this increased (18F)FDG uptake in the filling condition vs. empty condition to be located in the insular cortex. Clusters with decreased glucose uptake were detected in the cerebellum and the dorsal midbrain. Detailed cluster peak locations and *p* values of SPM findings are shown in Table 1 and Fig. 2. No other clusters were detected.

**Table 1:** Peak locations for the clusters in voxel-based analysis (at *p*_height_ < 0.005 uncorrected, *k*_E_ > 200) in two-sample *t* test for volume-induced voiding (*n* = 10) vs. empty bladder (*n* = 10)

Contrast	Voxel level		Structure			Name
	*T*	*p*_uncorr_	*x*	*y*	*z*	
VIV > EB	5.11	<0.001	2.8	2.4	−6.0	Insular cortex
VIV < EB	5.23	<0.001	0.4	−11.6	−5.4	Cerebellum
	4.72	<0.001	−2.0	−12.8	−5.6	Cerebellum
	3.73	0.001	−2.2	−8.0	−3.2	Dorsal midbrain

*VIV* volume-induced voiding, *EB* empty bladder

### Brain glucose metabolism during isovolumetric bladder contractions

To test whether isovolumetric bladder contractions provoke supraspinal metabolic changes, we compared brain glucose metabolism between the isovolumetric bladder contraction group and its control group. Predefined VOI analysis showed a significant glucose hypermetabolism in the temporal cortex in the isovolumetric contraction condition when compared to the empty bladder condition (+6.3 ± 2.3 %, *p* = 0.019). Uptake was decreased in the right cerebellum (−6.1 ± 1.6 %, *p* = 0.005). As illustrated in Table 2 and Fig. 2, voxel-based analysis confirmed the increased (18F)FDG uptake in an insular cluster and a smaller cluster located in the cingulate cortex. A cluster with decreased (18F)FDG uptake was again detected in the right cerebellar hemisphere (Fig. 2). No other clusters were detected.

**Table 2:** Peak locations for the clusters in voxel-based analysis (at *p*_height_ < 0.005 uncorrected, *k*_E_ > 200) in two-sample *t* test for isovolumetric contraction (*n* = 6) vs. empty bladder (*n* = 6)

Contrast	Voxel level		Structure			Name
	*T*	*p*_uncorr_	*x*	*y*	*z*	
IC > EB	3.86	0.002	6.8	−2.4	−6.6	Insular cortex
	3.10	0.005	0.8	0.8	−2.2	Cingulate cortex^a^
IC < EB	3.62	0.002	−1.2	−13.0	−4.0	Cerebellum

*EB* empty bladder, *IC* isovolumetric contraction

^a^Detected at *k*_E_ > 100

## Discussion

Since technological advancements have made it possible to study the supraspinal control of the bladder and urethra using PET and fMRI, increasing interest has been focused on the brain control of the LUT and its relationship with LUTd. The use of these imaging modalities in small laboratory animals allows a variety of experimental designs, which are not feasible in humans, and can help us to better understand the supraspinal control of the micturition cycle.

In this feasibility study, we have established a small-animal (18F)FDG-PET protocol to study brain control of the LUT during volume-induced voiding as well as isovolumetric bladder contractions. In comparison to isovolumetric bladder contractions, volume-induced voiding is a more physiological condition since the urethra is left unobstructed. However, it has the disadvantage of being a combination of both bladder filling and voiding, which could not be separately analysed because of cumulative brain glucose uptake and limited temporal resolution. Isovolumetric bladder contractions have previously been used to study brain control in rats using fMRI [7, 8] and more likely represent maximal bladder afferent input into the central nervous system but are less physiological because of urethral obstruction due to the urethral ligation and bladder (over)distension.

We demonstrated that continuous bladder filling as well as bladder fullness in rats provokes changes in brain activation similar to the changes seen in previous rat and human brain imaging studies at large bladder volumes, namely activation of the insular and cingulate cortices [4]. Since functional brain imaging does not provide insights into the functions of the detected regions, their function can only be inferred from previous electrophysiological and neuroanatomical experiments. It is well known that bladder afferents relay in the periaqueductal grey (PAG) and thalamus and then pass to more distal regions such as the insular and anterior cingulate cortices [11]. These neurons represent many aspects of the physiological conditions of the tissues of the body [12]. Activation in both regions has already been detected during the micturition cycle in rodents using fMRI [7, 8]. Co-activation of the insular cortex and ACG is frequently detected in functional brain imaging studies [13] and in almost all LUT-related brain imaging studies in humans [14]. In women with normal bladder function, insular activation also becomes stronger at large bladder volumes and strong desire to void [4]. The ACG is also implicated in the sensation of bladder fullness and has been related to emotional and motivational aspects of micturition [15]. Among other functions, the ACG can be considered as the cortical region associated with motivation [16].

Our results also show a glucose hypometabolism in the cerebellum during both conditions (Tables 1 and 2 and Fig. 2). Cerebellar involvement in micturition control has been reported by several human brain imaging studies [17–19], but its exact role remains elusive. It has been suggested to play a role in the coordination of the pelvic floor muscles [20]. Involvement of a region in the dorsal midbrain (Table 1 and Fig. 2a) was also detected in a fMRI study in rats and could possibly be attributed to activation of nociceptive bladder afferent nerves since measurements of immediate early gene expression in the brain after bladder irritation with cyclophosphamide identified this region [21]. Nevertheless, this remains speculative since no such region has been detected in human brain imaging studies [7].

fMRI and PET brain imaging are both very complementary methods to study brain metabolic processes, each carrying its own advantages and disadvantages. PET requires the injection of a radioactive isotope that is concentrated in metabolic active brain regions: (18F)FDG is taken up by glucose transporters and trapped inside the cell as a result of phosphorylation [22]. fMRI measures the changing proportion of oxygenated and deoxygenated haemoglobin in activated brain areas. PET brain imaging is sensitive enough to detect delicate changes in brain metabolism since few receptors are needed to trigger specific accumulation of the imaging agent while fMRI requires repeated trials to increase signal-to-noise ratio [23]. PET imaging offers a practical advantage in this type of research: cystometric equipment can be placed directly adjacent to the scanner whereas this is not possible with fMRI because of the strong magnetic field. However, because of the accumulation of tracer in activated brain areas, the temporal resolution of PET is more limited than that of fMRI.

As the temporal resolution of (18F)FDG is very limited (half-life period of 110 min), we have averaged images during volume-induced voiding and isovolumetric bladder contractions. This averaging inevitably results in a convolution of multiple neural processes where information on brain regions of interest may be lost. However, because of the use of this specific technique and tracer, it is currently not possible to improve temporal resolution. (15O)-H_2_O, which has a shorter half-life period, has previously been used in human PET studies looking at brain control of the LUT. However, because of our limited experience with this tracer, the availability of (18F)FDG and its proven validity in animal studies [24], we preferred (18F)FDG as a metabolic tracer to conduct this study. Apart from the temporal resolution, also the spatial resolution of glucose PET imaging is limited because of the limited resolution of the tomograph and relatively small brain size of rats. In order to improve sensitivity, we averaged brain metabolic images. Because of the accumulation of tracer in metabolically active brain regions, this technique is also not suitable for repeated testing in the same animal. Preferably, two or more different groups of animals should be used to test different conditions. Another limitation of this study is the use of urethane anaesthesia. Although it seems the best available anaesthetic to maintain the micturition response, it impairs bladder function [25]. On the other hand, anaesthesia minimizes the influences of other brain processes such as attention and emotion that also very likely influence voiding function.

Other studies also provided evidence for other brain regions to be involved in the neural control of the LUT, such as the periaqueductal grey, pontine micturition centre, thalamus, hypothalamus, amygdala and basal ganglia [14]. Two studies already described brain activation during cystometry in rats [7, 8]. Tai et al. showed the activation of similar brain areas during storage and voiding (i.e. insular and cingulate cortices). Wong et al. also described activation of the insular cortex during reflexive micturition. None of these studies looked at areas of deactivation. Also, uni- or bilateral activation of brain areas was reported by several human studies. However, in this study, we only found unilateral activation and deactivation of the brain areas, as described above. In general, interspecies variability (human vs. rat), differences in imaging techniques ((18F)FDG- or (15O)H_2_O-PET vs. fMRI), experimental setup (bladder filling and/or voiding), technical aspects of the scanner (spatial and temporal resolution), different analysis protocols and the use of anaesthesia (urethane) potentially influence the outcome of functional brain imaging and thus can account for these apparent differences between brain imaging studies of LUT control.

Taken together, our results demonstrate that it is feasible to study LUT-related brain metabolism in a rat model during cystometry using (18F)FDG-PET. Moreover, our findings are complementary with previous neuroanatomical and electrophysiological studies and human and rat brain imaging studies [7, 8, 19, 26].

## Conclusions

In this feasibility study, we show that (18F)FDG-PET brain imaging is a valuable technique to study the supraspinal control of the LUT in rats. It is complementary to fMRI in small laboratory animals, technically less challenging and very sensitive to detect small metabolic neural changes. We demonstrate that volume-induced voiding as well as bladder fullness in rats provokes changes in brain metabolism similar to the changes detected in humans, including activation of the insular and cingulate cortices, which is consistent with their role in the mapping of bladder afferent activity. Our results provide a basis for further research into the brain control of the LUT in small laboratory animals.

## Abbreviations

(18F)FDG-PET: fluorodeoxyglucose positron emission tomography
ACG: anterior cingulate cortex
fMRI: functional magnetic resonance imaging
LUT: lower urinary tract
LUTd: lower urinary tract dysfunction
PAG: periaqueductal grey
PMC: pontine micturition centre
SPM12: Statistical Parametric Mapping 12
SUV: standard uptake values
VOI: volume of interest
